# Association of sleep duration and sleep quality with overweight/obesity among adolescents of Bangladesh: a multilevel analysis

**DOI:** 10.1186/s12889-022-12774-0

**Published:** 2022-02-21

**Authors:** Md Rifat Anam, Shamima Akter, Fahima Hossain, Sharmin Quazi Bonny, Jahanara Akter, Cherri Zhang, Md. Mizanur Rahman, Md. Abul Basher Mian

**Affiliations:** 1 Global Public Health Research Foundation, Dhaka, Bangladesh; 2 National Center for Global Health and Medicine, Department of Epidemiology and Prevention, Tokyo, Japan; 3 Hitotsubashi Institute for Advanced study, Hitotsubashi University, 2-1 Naka Kunitachi, 186-8601 Tokyo, Japan; 4 Department of Psychology, University of Calgary, Calgary, AB Canada

**Keywords:** Sleep duration, Sleep quality, Adolescents, Overweight, Obesity, Bangladesh

## Abstract

**Background:**

Sleep deprivation is widely recognized as a potential contributor to childhood obesity. However, few studies have addressed this issue in low-income settings. The aim of this study was to determine the association of both sleep duration and sleep quality with overweight/obesity among adolescents of Bangladesh.

**Methods:**

A cross-sectional study was conducted in four randomly selected schools in Gazipur, Bangladesh, from May to August 2019. Using a self-administered semi-structured questionnaire, data on sleep duration and sleep quality were collected from 1,044 adolescents between 13 and 17 years of age. The body mass indices of the study participants were evaluated using their objectively-assessed anthropometric measurements (weight and height). Multilevel logistic regression was used for data analysis.

**Results:**

The prevalence of underweight, overweight and obesity in adolescents in this study were 14.9, 18 and 7.1%, respectively. More than 15% of the students reported sleep disturbance and poor sleep quality. After adjusting for confounders, reduced (<7 h/day) total sleep duration (OR=1.73, 95% CI=1.21-2.47), weekend sleep duration (OR=1.46, 95% CI=1.00-2.12), and night sleep duration (OR=1.55, 95% CI=1.06-2.28) were found to be significantly associated with overweight or obesity in Bangladeshi adolescents. Similarly, significant positive associations were evident between short duration of total sleep (OR=0.33, 95% CI=0.20-0.54), weekday sleep (OR=0.55, 95% CI=0.35-0.84), weekend sleep (OR=0.53, 95% CI=0.31-0.89), and night sleep (OR=0.56, 95% CI=0.36-0.87), and underweight in study participants. Adolescents with short sleep duration were found less likely to be underweight and more likely to be overweight/obese.

**Conclusions:**

Study findings denoted short sleep duration to be associated with overweight/obesity and underweight among adolescents of Bangladesh. Adequate sleep may therefore serve as an effective obesity prevention strategy in the growing stages.

**Supplementary Information:**

The online version contains supplementary material available at 10.1186/s12889-022-12774-0.

## Background

Worldwide, the rising prevalence of obesity is a major public health concern for both adults and adolescents [1]. The dramatic increase in pediatric obesity is contemplated an universal threat, not only because of its association with numerous co-morbidities, economic burden, and detrimental impacts on quality of life, but also due to its continued progression into adulthood obesity [2, 3]. The global rate of pediatric obesity rose from less than 1% (equivalent to five million girls and six million boys) in 1975 to nearly 6% for girls (50 million) and about 8% for boys (74 million) in 2016, indicating more kids and adolescents will have obesity by 2022 if present trends continue [4]. In a South Asian country like Bangladesh, where 36 million adolescents make up 22% of its population, similar trend of weight gain among the pediatric age group has been observed [5, 6].

Being a multifactorial condition, obesity results from inalterable genetic and potentially modifiable risk factors. The latter, involving human behavior and lifestyle modification, plays an extremely crucial role in developing obesity as periods of childhood and adolescence are vulnerable to developing habits that promote weight gain. Hence, other than ensuring sufficient physical activity and a healthy diet, recent literature exhibits growing interests in associating sleep inadequacy as a risk factor for obesity, especially among children and adolescents [7, 8]. Although little is known about the exact mechanism, sleep deprivation has been hypothesized to cause weight gain in adolescents through hormonal disruption, daytime sleepiness, and fatigue [9, 10]. In healthy individuals, sleep curtailment simultaneously elevates serum ghrelin and declines leptin levels, resulting in enhanced appetite and reduced satiety with an overall increase in hunger for calorie-dense high-carbohydrate containing foods [11]. In addition, sleep deprivation also causes an increase in cortisol level, insulin resistance, and impaired glucose tolerance, all of which affect weight status [12]. On the contrary, daytime sleepiness and fatigue cause exhaustion and inhibit participation in physical activities and healthy food preparation which eventually provide a barrier to active lifestyle [13].

In developed nations, short sleep duration has been well recognized as a potential contributor to pediatric obesity and demonstrated a negative linear association in several cross-sectional and longitudinal epidemiological studies [7, 8, 14–22]. From the resource-limited settings such as China [23] and India [24], studies illustrated similar significant association between the two variables. For quite some time, most studies utilized only sleep duration as the sole measure for inadequate sleep, which is a misleading approach since sleep is multidimensional [25]. Hence, satisfactory sleep experience must be defined in terms of both optimal sleep duration and sleep quality, and evaluated among the youth especially in a country like Bangladesh, which is undergoing rapid urbanization and industrialization. To date, only three studies in Bangladesh have assessed the association between total sleep duration and pediatric obesity, but none of them revealed a significant relationship between these two parameters and variations in night and day time sleep duration or weekend and weekday sleep duration were not considered [26–28]. In addition, no studies have determined the link between sleep quality and overweight/obesity among the Bangladeshi youth. Therefore, to the best of our knowledge, this is the first study of its kind which aims to determine the association of both sleep duration and sleep quality with overweight/obesity in the adolescents of Bangladesh.

## Methods

### Study design

A cross-sectional study was conducted among adolescent students (both boys and girls) from four randomly selected schools of Gazipur district in Bangladesh between May and August 2019. Two rural and two urban schools were chosen for the present study. Students were recruited from grades 8 and 9, after obtaining students’ assent and parental or legal guardian’s consent. A self-reported semi-structured questionnaire was used for data collection (Supplementary material). On the day of health checkup, trained enumerators distributed the questionnaires to the students at different classes of both grades and provided them with detailed instructions. Students signed the assent forms and filled out the questionnaires with the help of the assigned enumerators. After completion of the interviews, field supervisors checked the questionnaires for completeness and, where necessary, clarified responses with the participants. Ethical clearance was obtained from the National Center for Global Health and Medicine in Japan and the University of Rajshahi in Bangladesh. Formal approval from the participating school authorities, written parental consent, and adolescent assent were also obtained.

### Study subjects

Of the 1107 students in total, 1047 agreed to participate in the survey and completed the questionnaire with an overall response rate of 94.6%. The rest of the students were absent on the day of data collection. Amongst the 1047 respondents, aged 13 to 17 years from grades 8 and 9, some were excluded due to missing data in the variables sleep quality (*n *= 1) and age (*n *= 1), and being an outlier in the BMI variable (*n *= 1). After these exclusions, a total of 1044 students (505 boys and 539 girls) were finally eligible for the present study.

### Anthropometric measurements

Anthropometric variables included body weight and height measurements. Under the direct supervision of research assistants, trained data enumerators performed the measurements within the classroom according to written standardized procedures. Body weight was measured to the nearest 0.1 kg using calibrated portable scale with minimal clothing and no shoes. Height was measured to the nearest 0.1 cm using calibrated portable measuring rod with the participants in fullstanding position without shoes. Body mass index (BMI) was calculated using the following formula: person’s weight in kilograms divided by height in meters squared (kg/m^2^).

### Classification of weight status

To define the weight status of the participants in terms of underweight, normal weight, overweight and obesity, World Health Organization's (WHO) growth reference for 5-19 years was used [29]. As all the respondents were less than 18 years of age, they were classified into the following four categories on the basis of their BMI scores falling within the standardized value for age and sex- (a) underweight: BMI ≤-2SD of Z scores, (b) normal weight: BMI within the range of: >-2SD to +1SD of Z scores, (c) overweight: BMI >+1SD to +2SD of Z scores, and (d) obese: BMI >+2SD of Z scores.

### Sleep duration and sleep quality

Sleep duration of the participants wase recorded in hours from their answers to the following questions: “How many hours, approximately, do you usually sleep during a night?”, “How many minutes, approximately, do you usually nap during a day?”, “How many hours, approximately, do you usually sleep in a weekday?”, and “How many hours, approximately, do you usually sleep in a weekend?”. Total sleep duration each day included both daytime nap and nighttime sleep and was calculated by averaging weekdays' and weekend's sleep duration. Each variable of sleep duration (except for daytime nap duration) was categorized in the analysis as follows: ≥8, 7 to <8 and <7 h of sleep, adopted from the National Sleep Foundation recommendation on sleep time [30]. Since there was no particular consensus among sleep professionals as to what amount of naptime was adequate for different age groups, daytime nap duration for this study was categorized as follows: 0, ≤ 45 and >45 min, based on the findings of Sleep in America poll by the National Sleep Foundation in 2011 [31]. The poll findings concluded that adolescent respondents’ naptime lasted on an average of 43 min on weekdays and 46 min on weekends [31]. Weekday – weekend difference in sleep duration was categorized as -4 to <0 h, 0 to 1 h, >1 to 2 h, >2 to 3 h, >3 to 8 h, and the second category was used as the reference category as it represented the smallest absolute difference between weekday’s and weekend’s sleep.

Sleep disturbance was assessed by asking the question, “Do you face any sleep disturbance at night?” and the response was either “Yes” or “No”. We also assessed overall sleep quality using the following single question: “How was your sleep in the last one month?” and the response options were “1) bad, 2) not so good, 3) good, 4) very good”. In this study, we considered “not so good” and “bad” denoting poor sleep quality, whereas the responses of “good” and “very good” were considered as good sleep quality, based on a previous study [32].

### Covariates

Covariates including age (continuous), sex (male or female), number of household members (continuous), soft drink consumption (less than once/week, once/week, ≥2 times/week), fast food consumption (less than once/week, once/week, ≥2 times/week), general physical activity (<30 min/day, 30-59 min/day, ≥60 min/day), sedentary activities (TV watching, playing computer games etc., : <5 h a day, ≥ 5 h a day), and exposure to passive smoking (0 days, 1-2 days, 3-4 days, ≥5 days) were assessed via a standard questionnaire.

### Statistical analysis

Descriptive statistics were presented as means, standard deviations and proportions. Multilevel logistic regression models were used to examine the relationship between sleep duration and sleep quality and the state of being underweight, overweight or obese. To adjust for the clustering effect of schools, when analyzing obesity status according to sleep duration and sleep quality at the individual level, we used a two-level regression model with a school-level random intercept. Two different multilevel models were considered. In the first model, we adjusted for age (continuous) and sex (male or female). In the second model, we further adjusted for the number of household members (continuous), soft drink consumption (less than once/week, once/week, ≥2 times/week), fast food consumption (less than once/week, once/week, ≥2 times/week), general physical activity (<30 min/day, 30-59 min/day, ≥60 min/day), sitting activities (<5 h a day, ≥ 5 h a day) and exposure to passive smoke (0 days, 1-2 days, 3-4 days, ≥5 days). Data were analyzed using Stata, version 14.2 (Stata Corporation, College Station, TX, USA).

## Results

The descriptive characteristics of the participants are shown in Table 1. The study sample included more girls than boys (51.6% vs. 48.4%) whose mean ages were 13.72 ± 0.87 and 14.01 ± 0.97 years, respectively. The mean BMI of all the respondents was 20.58 ± 4.6 kg/m^2^. The prevalence of underweight, overweight and obesity in the Bangladeshi adolescents were 14.9, 18 and 7.1%, accordingly. The average sleep duration for each day was 7.61 ± 1.33 h and the average weekdays and weekend sleep durations were 6.78 ± 1.25 and 7.81 ± 1.51 h, respectively. The mean per night sleep duration was 7.07 ± 1.22 h and daytime nap was 32.15 ± 41.69 min (median 17.14 min). Almost one-third (31%) of the respondents had less than 7 h of total sleep with majority sleeping less than 7 h at night (46.3%). More than half of the respondents (51.44%) had daytime naps, where one-fourth (26.44%) slept for more than 45 min. In addition, shorter sleep duration was more prevalent on weekdays than on weekends. 15.3% of the participants reported having bad sleep quality and sleep disturbance was prevalent among almost all of them.

**Table 1:** Sample characteristics of adolescents from Bangladesh

Characteristics	Frequency (*N* = 1044)	Percentage (%)
**Gender**		
Boys	505	48.4
Girls	539	51.6
**Total sleep**		
<7	324	31.0
7 to <8	315	30.2
≥8	405	38.8
**Night sleep**		
<7	483	46.3
7 to <8	291	27.9
≥8	270	25.9
**Weekday sleep**		
<7	492	47.1
7 to <8	287	27.5
≥8	265	25.4
**Weekend sleep**		
<7	209	20.0
7 to <8	223	21.4
≥8	612	58.6
**Nap (mins)**		
0	507	48.56
<=45	261	25.00
>45	276	26.44
**Sleep disturbance**		
Yes	159	15.2
No	885	84.8
**Sleep quality**		
Good	884	84.7
Bad	160	15.3
**BMI Status**^**a**^		
Underweight	155	14.9
Normal	626	60.1
Overweight	187	18.0
Obese	74	7.1

^a^*BMI*  Body Mass Index

Moreover, there was a strong positive correlation between weekday sleep duration and weekend sleep duration (*r *= 0.59). As weekday sleep duration increased, weekend sleep duration also increased in this study. A weakly negative correlation between night sleep duration and daytime nap duration (*r *= -0.12) was also found. As night sleep duration decreased, daytime nap increased in these respondents.

Table 2 represents the association between sleep duration and the prevalence of underweight and overweight/obesity in the study subjects. Model 1 exhibited that short durations of total sleep (<7 h/d; OR = 1.71, 95% CI=1.21 to 2.42, *p*-value=0.002), weekend sleep (<7 h/d; OR=1.48, 95% CI=1.03 to 2.12, *p*-value=0.018), night sleep (<7 h/d; OR=1.56, 95% CI=1.06 to 2.26, *p*-value=0.019), and daytime sleep (0 min/day; OR=1.45, 95% CI=1.01 to 2.08, p-value=0.047) to be significantly and inversely associated with overweight/obesity. In model 2, with adjustment for lifestyle-related covariates, those who slept for less than 7 h a day at night and in total were associated with 1.55 and 1.73 higher odds of overweight/obesity, respectively (Supplementary Table S1, Table S2, and Table S3). The findings are almost similar for weekend night sleep duration (OR=1.46, 95% CI=1.00 to 2.12, *p*-value=0.029) (Supplementary Table S4).

In model 1, compared to long duration (≥8 h/d), short total sleep duration (<7 h/d) was found to be significantly associated with a decreased prevalence of underweight in Bangladeshi adolescents (OR= 0.33, 95% CI: 0.20 to 0.53). Also, in model 2, after adjustment for lifestyle-related covariates, a dose-response relationship was observed between total sleep duration and underweight in this study sample (*p*-trend=<0.001). Short weekday sleep duration (<7 h/d) was also significantly associated with a decreased prevalence of underweight in model 2 (OR=0.55, 95% CI: 0.35 to 0.84) as compared with long sleep duration (≥8 h/d) after additional adjustment for lifestyle covariates. Similarly, a dose-response relationship was also observed between weekend sleep duration and underweight among adolescents, from the lowest (<7 h/d) to the highest (≥8 h/d) category of weekend sleep duration which were 0.53 (0.31 to 0.89), 0.79 (0.50 to 1.25), and 1.00 (reference), respectively (*p*-trend=0.02) in model 2. Short night sleep duration (<7 h/d) compared to long sleep duration (≥8 h/d) was found to be significantly associated with a decreased prevalence of underweight after additional adjustment for lifestyle covariates in model 2 (OR= 0.56, 95% CI: 0.36 to 0.87; *p*-trend=0.008). Likewise, those who did not sleep during the day were significantly and inversely associated with a decreased prevalence of underweight in model 2 (OR= 0.56, 95% CI: 0.37 to 0.85; *p*-trend=0.010). Findings were almost similar for both weekday and weekend night sleep duration [OR=0.55 (0.35 to 0.84), *p*-trend=0.006 and OR=0.53 (0.32 to 0.89), *p*-trend=0.015, respectively] (Supplementary Table S4).

For weekday – weekend difference in sleep duration, we found no significant association of this difference with overweight/obesity and underweight both in models 1 and 2 (Table 2).

In addition, we examined the association between covariates and obesity status (underweight, normal, and overweight/obese) (Supplementary Table S5) and found significant difference only between age and household members with obesity status.

**Table 2:** Multivariable-adjusted odds ratio and 95% confidence interval for the association of sleep duration with underweight and overweight/obesity

	Underweight		Overweight/obesity		
	Model 1^a^	Model 2^b^	Model 1^a^	Model 2^b^	
**Total sleep duration (hours/day)**					
≥8	1.00 (reference)	1.00 (reference)	1.00 (reference)	1.00 (reference)	
7 to <8	0.75 (0.51-1.13)	0.76 (0.50-1.15)	1.11 (0.77-1.59)	1.10 (0.76-1.60)	
<7	0.33 (0.20-0.53)	0.33 (0.20-0.54)	1.71 (1.21-2.42)	1.73 (1.21-2.47)	
* P*_*trend*_	<0.001	<0.001	0.002	0.003	
**Weekday sleep duration (hours/day)**					
≥8	1.00 (reference)	1.00 (reference)	1.00 (reference)	1.00 (reference)	
7 to <8	0.77 (0.49-1.21)	0.77 (0.49-1.21)	1.19 (0.79-1.80)	1.17 (0.77-1.78)	
<7	0.54 (0.35-0.82)	0.55 (0.35-0.84)	1.43 (0.98-2.09)	1.43 (0.98-2.11)	
* P*_*trend*_	0.004	0.006	0.056	0.058	
**Weekend sleep duration (hours/day)**					
≥8	1.00 (reference)	1.00 (reference)	1.00 (reference)	1.00 (reference)	
7 to <8	0.79 (0.50-1.23)	0.79 (0.50-1.25)	1.42 (0.99-2.02)	1.36 (0.95-1.96)	
<7	0.53 (0.32-0.89)	0.53 (0.31-0.89)	1.48 (1.03-2.12)	1.46 (1.00-2.12)	
* P*_*trend*_	0.01	0.02	0.018	0.029	
**Night sleep duration (hours/day)**					
≥8	1.00 (reference)	1.00 (reference)	1.00 (reference)	1.00 (reference)	
7 to <8	0.88 (0.57-1.38)	0.90 (0.58-1.42)	1.24 (0.82-1.88)	1.21 (0.80-1.85)	
<7	0.56 (0.36-0.85)	0.56 (0.36-0.87)	1.56 (1.06-2.26)	1.55 (1.06-2.28)	
* P*_*trend*_	0.006	0.008	0.019	0.020	
**Daytime nap duration (minutes/day)**					
>45	1.00 (reference)	1.00 (reference)	1.00 (reference)	1.00 (reference)	
≤ 45	0.61 (0.38-0.97)	0.57 (0.35-0.93)	1.30 (0.86-1.97)	1.41 (0.93-2.15)	
0	0.56 (0.38-0.84)	0.56 (0.37-0.85)	1.45 (1.01-2.08)	1.52 (1.05-2.19)	
* P*_*trend*_	0.007	0.010	0.047	0.034	
**Difference between weekday and weekend sleep duration (hours)**					
-4 to <0	1.03 (0.62-1.72)	1.04 (0.62-1.74)	1.46 (0.98-2.18)	1.46 (0.97-2.18)	
>0 to 1	1.00 (reference)	1.00 (reference)	1.00 (reference)	1.00 (reference)	
>1 to 2	1.24 (0.74-2.07)	1.25 (0.74-2.10)	1.01 (0.66-1.54)	1.06 (0.69-1.63)	
>2 to 3	1.19 (0.64-2.21)	1.19 (0.63-2.23)	1.00 (0.60-1.66)	1.05 (0.63-0.87)	
>3 t0 8	1.60 (0.84-3.04)	1.57 (0.81-3.02)	0.62 (0.33-1.15)	0.60 (0.32- 1.14)	

^a^Model 1 adjusted for age (year, continuous) and sex (boys or girls)

^b^Model 2 variables adjusted in model 1 + number of household members (continuous), soft drink consumption (less than once/week, once/week, ≥2 times/week), fast food consumption (less than once/week, once/week, ≥2 times/week), general physical activity (<30 min/day, 30-59 min/day, ≥60 min/day), sitting activities (<5 h a day, ≥ 5 h a day), and exposure to passive smoking (0 days, 1-2 days, 3-4 days, ≥5 days)

Table 3 presents the association between sleep quality and the prevalence of underweight and overweight/obesity in the study subjects. Overall, we found no significant association between sleep disturbance and sleep quality with neither overweight/obesity nor underweight adolescents.

**Table 3:** Multivariable-adjusted odds ratio and 95% confidence interval for the association of sleep quality with underweight and overweight/obesity

	Underweight		Overweight/obesity	
	Model 1^a^	Model 2^b^	Model 1^a^	Model 2^b^
**Sleep disturbance**				
No	1.00 (reference)	1.00 (reference)	1.00 (reference)	1.00 (reference)
Yes	0.95 (0.58-1.57)	0.94 (0.56-1.57)	0.84 (0.56-1.26)	0.85 (0.56-1.29)
**Sleep quality**				
Good	1.00 (reference)	1.00 (reference)	1.00 (reference)	1.00 (reference)
Bad	1.41 (0.90-2.23)	1.38 (0.86-2.20)	0.68 (0.44-1.05)	0.70 (0.46-1.09)

^a^Model 1 adjusted for age (year, continuous) and sex (boys or girls)

^b^Model 2 variables adjusted in model 1 + number of household members (continuous), soft drink consumption (less than once/week, once/week, ≥2 times/week), fast food consumption (less than once/week, once/week, ≥2 times/week), general physical activity (<30&nbsp;min/day, 30-59&nbsp;min/day, ≥60&nbsp;min/day), sitting activities (<5&nbsp;h a day, ≥ 5&nbsp;h a day), and exposure to passive smoking (0 days, 1-2 days, 3-4 days, ≥5 days)

## Discussion

The key findings of this cross-sectional study denote short sleep duration to be significantly associated with a higher prevalence of overweight/obesity and a conversely lower prevalence of underweight in the adolescents of Bangladesh. To the best of our knowledge, it is the first study to investigate the association between weekday and weekend sleep durations and sleep quality with overweight or obese adolescents in South Asia. In addition, it is the first study of its kind to investigate the effects of both sleep duration and sleep quality on the risk of being overweight or obese among Bangladeshi adolescents.

The mean sleep duration among adolescents found in this study from Bangladesh (7.61 h/day) is relatively similar to most reports from other Asian countries such as India (7.8 h/day) [33], Taiwan (7.35 h/weekday) [34] and China (7.5 h/weekday) [35]. Relevant studies from high-income countries like Germany (7.8 h/day) [36], Switzerland (7.9 h/day) [37], South Africa (7.55 h/weekday) [38] and Saudi Arabia (7.2 h/day) [39] also exhibited similar findings, with few exceptions observed among the Spanish adolescents (8.35 h/day) [40] and a study from ten European cities (8 h/day) [41].

The current study illustrated total sleep duration to be inversely associated with overweight or obesity among Bangladeshi adolescents which is not consistent with the three previous studies conducted in Bangladesh [26–28]. The probable reasons for not finding any significant relationship between these two variables in those studies may be due to the absence of adjusting potential confounders, recruitment of respondents only from urban schools, consideration of only total sleep duration as an independent variable, and selection of relatively smaller sample sizes (*n*=<300). However, the present findings are consistent with some studies from Asian countries like China [23] and India [24] that reported significant association between sleep duration and overweight/obesity. The Indian study signified inadequate sleep duration with higher odds of adolescents being overweight or obese (OR=1.56, 95% CI=1.12 -2.15), [24] whereas the Chinese study found a U-shaped association between sleep duration and the risk of obesity among their youths [23]. Several studies conducted in some developed countries such as in Turkey, [42] Saudi Arabia, [43] USA, [18] Portugal, [19] Australia [20] and Canada [21] also reported significant inverse associations between sleep duration and overweight or obesity. The present study not only confirmed the previous findings but also extended the evidence to low-income settings such as Bangladesh, suggesting that short sleep duration increases the risk of overweight or obesity in adolescents.

In addition, the present study has demonstrated a noteworthy association between weekend sleep duration and overweight/obesity development in adolescents which is in line with the findings from China [44] and Korea [45]. Both studies emphasized on the fact that children with shorter sleep duration during weekdays have significantly higher chances of being overweight or obese if they do not compensate for their sleep deficits adequately during the weekends. Similarly, our study exhibited a significant relationship between night sleep duration and overweight/obesity development which is similar to the finding of a previous study from Saudi Arabia [39].

All in all, may it be day or night, weekday or weekend, short sleep duration at any time has a remarkable influence on the weight status of growing children especially adolescents as it is a crucial period of life for obtaining autonomy, enhancing socialization, and developing a burning desire to adapt the modern lifestyle. Adding to these is the immense pressure from school for increased study load and home tasks – all of which cumulatively result in sleep curtailment, tiredness, reduced time for physical activities and eventual consumption of energy-dense foods in the additional wake times among the youth. These societal and behavioral factors seem to notably complement the previously hypothesized mechanisms within the body, such as hormonal dysregulation, metabolic pathway alteration, daytime sleepiness and fatigue, [9–13] through which sleep deprivation ultimately results in higher energy consumption, lower energy expenditure, adoption of unhealthy behavioral lifestyle, and eventual weight gain.

In the present study, the prevalence of underweight was found to be 14.9%, which is in line with a previous study in Bangladesh [46]. Short total sleep duration has been found to have a noteworthy relationship with the decreased odds of underweight. Although the research primarily focused on the effect of sleep on overweight or obesity in adolescents, the aforementioned association between underweight and short sleep duration was quite interesting. This finding is consistent with a relevant study from Australia [47] where, short sleep duration was significantly associated with weight status category, with sleep duration decreasing linearly from underweight through obese [47]. That is, underweight adolescents substantially slept longer than the overweight and obese adolescents [47]. Even though not much is known about the possible pathways and mechanisms for this association, literature suggests that a low-calorie intake can result in sleep fragmentation and reduction of slow wave sleep [48]. Furthermore, it can also be responsible for declining the sleep inducing gut peptides (i.e. cholecystokinin) as well as increasing the levels of wake agents such as orexin [48]. For weekday sleep duration, weekend sleep duration, night sleep duration, daytime nap duration, weekday night sleep duration, and weekend night sleep duration a similar trend is observed in the underweight participants of the present study. A study conducted on Norwegian adolescents exhibited a significant association between weekday short sleep duration and being underweight, which supports our study finding [22]. Although the study also stated no significant association between weekend short sleep duration and underweight, [22] our study denoted a significant association between the two variables. The possible reason for this association could be attributed to eating fewer calories by skipping meals while sleeping during the weekends [49]. More experimental or prospective research is required to explore how Bangladeshi adolescents with food insecurity and hunger may feel lethargic and sleepy and hence spend more time sleeping, since the country is experiencing a dual burden of both under- and over-nutrition and their respective health hazards need to be addressed urgently with great emphasis.

The major strength of this study is it takes into consideration the four different timings of sleep hours (weekday, weekend, daytime, and nighttime) among the adolescent age group. With few exceptions, [44, 45] most previous investigations focused primarily on the mean sleep duration throughout the week to determine the weight changes in the growing period of life. Also, no previous study in Bangladesh and very few studies worldwide have included sleep duration and sleep quality together to assess their effects on weight gain in adolescence [26–28, 50]. Another strength of the present study includes the adjustment for potential confounding variables such as physical activity, sedentary behaviors, and some dietary intake variables.

This study has certain limitations. First, in a cross-sectional study, an observed association does not imply causal association. Second, sleep duration and sleep quality were estimated by a self-reported questionnaire, which could be prone to recall bias and might not provide accurate information about sleep pattern. Moreover, in this study, the participants’ sleep quality was assessed by only two questions, one of which was a single-item question focusing on their previous month’s experience, which could be prone to both recall bias and other probable unmeasured residual confounders (e.g., conditions of mood and personality of the respondents at the time of data collection), and therefore be the likely reason for the insignificant associations observed with the different weight statuses. Future studies are warranted for further detailed evaluation of these sleep measures while keeping the observations of this study in perspective. Third, no information was collected on parental education or income, hence we were unable to control for or assess the joint effect of these factors or socio-economic status on overweight or obesity among children. Finally, data were collected from students of four schools of Gazipur district, Dhaka. Therefore, present findings might not be generalizable to all adolescents in Bangladesh.

## Conclusions

Sleep inadequacy is prevalent among adolescents of Bangladesh. Short sleep duration has a significant positive association with overweight/obesity. Adequate sleep is mandatory for the adolescents of Bangladesh to reduce the burden of both pediatric and adult obesities. Further large-scale studies are needed to assess all sleep dimensions and confirm the present findings.

## Supplementary information

**Additional file 1. **Student questionnaire

## Data Availability

The datasets generated and/or analyzed during the current study are not publicly available because we did not seek consent from participants to share the data publicly. However, the dataset is available from the corresponding author on a reasonable request.

## Abbreviations

BMI: Body Mass Index
WHO: World Health Organization
SD: Standard Deviation
