# Supplementary material for: Association of sleep duration and sleep quality with overweight/obesity among adolescents of Bangladesh: a multilevel analysis
Source: BMC Public Health. 2022 Feb 21;22:374. doi: 10.1186/s12889-022-12774-0 (PMC8862335; doi:10.1186/s12889-022-12774-0)
Supplement: Supplementary file 1 — Additional file 1. Student questionnaire [file 12889_2022_12774_MOESM1_ESM.docx]

**Title:** Association of sleep duration and sleep quality with overweight/obesity among adolescents of Bangladesh: a multilevel analysis

*Md Rifat Anam^a^, Shamima Akter ^a, b, c*^, Fahima Hossain^a^, Sharmin Quazi Bonny^a^, Jahanara Akter^a^, Cherri Zhang^a,d^, Md. Mizanur Rahman^c^, Md. Abul Basher Mian^a^*

^a^Global Public Health Research Foundation, Dhaka, Bangladesh

^b^National Center for Global Health and Medicine, Department of Epidemiology and Prevention,Tokyo, Japan

^c^Hitotsubashi Institute for Advanced study, Hitotsubashi University, Japan

^d^Department of Psychology, University of Calgary, Calgary, AB, Canada

**Authors:**

Md Rifat Anam (email: md.rifatanam@gmail.com)

(ORCID: https://orcid.org/0000-0001-7450-6977)

Shamima Akter (email: samimarub@yahoo.com)

Fahima Hossain (email: fht017@gmail.com)

Sharmin Quazi Bonny (email: bonny.sharminq@gmail.com)

Jahanara Akter (email: jahanaraju1978@gmail.com)

Cherri Zhang (email: toki.cherri.zhan28@gmail.com)

Md. Mizanur Rahman (email: mizanurrub78@gmail.com)

Md. Abul Basher Mian (email: director.gphrf@gmail.com)

**^*^Corresponding author:**

Shamima Akter, Hitotsubashi Institute for Advanced study, Hitotsubashi University, Japan

Tokyo 162-8655, Japan. Tel: +81-3-3202-7181, Fax: +81-3-3202-7364, E-mail: samimarub@yahoo.com

***Student Questionnaire***

|  |
| --- |

| \| **Step 1 Socio-demographic information** \| \| \| \| \| --- \| --- \| --- \| --- \| \| **SL** \| **Questions** \| **Response** \| **Code** \| \| 1 \| What is your name? \|  \| I1 \| \| 2 \| What is your father’s name? \|  \| I2 \| \| 3 \| What is your mother’s name? \|  \| I3 \| \| 4 \| In which grade/class are you in? \|  \| I4 \| \| 5 \| Sex \| 1= Male 2= Female \| I5 \| \| 6 \| What is your date of birth? \| Days…… Month……. Year…….. \| I6 \| \| 7 \| How old are you? \| Years………….. \| I7 \| \| 8 \| Total household members \|  \| I8 \| |
| --- | --- | --- | --- | --- | --- | --- | --- | --- | --- | --- | --- | --- | --- | --- | --- | --- | --- | --- | --- | --- | --- | --- | --- | --- | --- | --- | --- | --- | --- | --- | --- | --- | --- | --- | --- | --- | --- | --- | --- | --- |

| \|  \| **Step 2 Tobacco products** \| \| \| \| --- \| --- \| --- \| --- \| \| **SL** \| **Questions** \| **Response** \| **Code** \| \| 1 \| Does any member of your family smoke tobacco? \| 1= Yes 2= No  *[If* ***No*** *go to T4]* \| T1 \| \| 2 \| During past 30 days, Did your parents smoke inside your house? \| 1= Yes 2= No \| T2 \| \| 3 \| During past 7 days, how many days you have been exposed to tobacco smoking? \| 1= 0 days  2= 1 or 2 days  3=3 or 4 days  4=5 or 6 days  5=All 7 days \| T3 \| \| 4 \| Did any members of your family use any other form tobacco products such as *chewing tobacco, betel* etc. \| 1= Yes 2= No \| T4 \| \| 5 \| Have you ever tried smoking? \| 1= Yes 2= No \| T5 \| |
| --- | --- | --- | --- | --- | --- | --- | --- | --- | --- | --- | --- | --- | --- | --- | --- | --- | --- | --- | --- | --- | --- | --- | --- | --- | --- | --- | --- | --- |

| \| **Step 3 Dietary habits** \| \| \| \| \| --- \| --- \| --- \| --- \| \| 1 \| In a typical week, on how many days do you **eat fruit** such as bananas, guava, mango,pineapple, apples, oranges, jackfruit, boroi, or amra? \| No. of days: \| D1 \| \| 2 \| How many servings of fruit do you eat on one of those days \| Servings: \| D2 \| \| 3 \| In a typical week, on how many days do you **eat vegetables** such as tomato, cauliflower, eggplant, ladies finger, leafy vegetables**?** \| No. of days: \| D3 \| \| 4 \| How many servings of vegetables do you eat on one of those days? \| Servings: \| D4 \| \| 5 \| In a typical week, on how many days do you **eat white meat** such as chicken, duck, or pigeon? \| No. of days: \| D5 \| \| 6 \| How many servings of white meat do you eat on one of those days? \| Servings: \| D6 \| \| 7 \| In a typical week, on how many days do you **eat red meat** such as beef? \| No. of days: \| D7 \| \| 8 \| How many servings of red meat do you eat on one of those days? \| Servings: \| D8 \| \| 9 \| In a typical week, on how many days do you **eat fish**? \| No. of days: \| D9 \| \| 10 \| How many servings of fish do you eat on one of those days? \| Servings: \| D10 \| \| 11 \| In a typical week, on how many days do you **have milk or milk products including yogurt, kheer**? \| No. of days: \| D11 \| \| 12 \| How many servings of milk or milk products do you eat on one of those days? \| Servings: \| D12 \| \| 13 \| In a typical week, on how many days do you **eat Dal such as lentils, mung bean, chick pea, masur dal or motor dal**? \| No. of days: \| D13 \| \| 14 \| How many servings of dal do you eat on one of those days? \| Servings: \| D14 \| \| 15 \| In a typical week, on how many days do you eat cereals including **rice, ruti, porota, bread, chira, muri, oats, cornflakes, noodles**? \| No. of days: \| D15 \| \| 16 \| How many servings of cereals do you eat on one of those days? \| Servings: \| D16 \| \| 17 \| In a typical week, on how many days do you eat **Egg**? \| No. of days: \| D17 \| \| 18 \| How many servings of egg do you eat on one of those days? \| Servings: \| D18 \| \| 19 \| In a typical week, on how many days do you **eat fast foods such as hamburger, French fry etc.**? \| No. of days: \| D19 \| \| 20 \| In a typical week, how *many days* did you usually drink carbonated soft drinks, such as Coke, Fanta Orange, or 7-Up? \| No. of days: \| D20 \| |
| --- | --- | --- | --- | --- | --- | --- | --- | --- | --- | --- | --- | --- | --- | --- | --- | --- | --- | --- | --- | --- | --- | --- | --- | --- | --- | --- | --- | --- | --- | --- | --- | --- | --- | --- | --- | --- | --- | --- | --- | --- | --- | --- | --- | --- | --- | --- | --- | --- | --- | --- | --- | --- | --- | --- | --- | --- | --- | --- | --- | --- | --- | --- | --- | --- | --- | --- | --- | --- | --- | --- | --- | --- | --- | --- | --- | --- | --- | --- | --- | --- | --- | --- | --- | --- |

| \| **Step 4** **Physical activities** \| \| \| \| \| --- \| --- \| --- \| --- \| \| 1 \| During the past 7 days, on how many hours were you physically active per day? \| Duration: \| P1 \| \| 2 \| During the past 7 days, on how many days did you walk or ride a bicycle? \| No. of days: \| P2 \| \| 3 \| In a typical week, on how many **days** do you do **sports, fitness or recreational (leisure)** activities? \| No. of days: \| P3 \| \| 4 \| How **much time** do you spend doing **sports, fitness or recreational** activities on a typical day? \| Duration: \| P4 \| \| 5 \| In a typical week, how many days do you watch television? \| No. of days: \| P5 \| \| 6 \| How many hours do you spend watching television on those days? \| Duration: \| P6 \| \| 7 \| In a typical week, how many days do you handle electronic device such as computer, smart phone or tab? \| No. of days \| P7 \| \| 8 \| How many hours do you spend on electronic devices on those days? \| Duration: \| P8 \| \| 9 \| During the past 30 days, on *how many days* did you *miss classes or school* without permission? \| No. of days: \| P9 \| |
| --- | --- | --- | --- | --- | --- | --- | --- | --- | --- | --- | --- | --- | --- | --- | --- | --- | --- | --- | --- | --- | --- | --- | --- | --- | --- | --- | --- | --- | --- | --- | --- | --- | --- | --- | --- | --- | --- | --- | --- | --- |

| \| **Step 5 Sleep** \| \| \| \| \| --- \| --- \| --- \| --- \| \| 1 \| On a week day, how many **hours** do you sleep at **NIGHT**? \| Duration: \| S1 \| \| 2 \| On a week day, how many **hours** do you sleep during the day time? \| Duration: \| S2 \| \| 3 \| On a weekend, how many **hours** do you sleep at **NIGHT**? \| Duration: \| S3 \| \| 4 \| On a weekend, how many **hours** do you sleep during the day time? \| Duration: \| S4 \| \| 5 \| Do you face any sleep **disturbance** at **night**?  *[Make circle appropriate answer]* \| 1= Yes  2= No \| S5 \| |
| --- | --- | --- | --- | --- | --- | --- | --- | --- | --- | --- | --- | --- | --- | --- | --- | --- | --- | --- | --- | --- | --- | --- | --- | --- |

| \| **Step 6 Risk/Awareness questionnaire** \| \| \| \| \| --- \| --- \| --- \| --- \| \|  \| **Questions** \| **Response** \| **Code** \| \| 1 \| When you were in school, were you taught about the dangers of smoking (for example, lung cancer, heart disease, fire hazards)? \| 1= Yes 2= No \| R1 \| \| 2 \| When you were in school, were you taught about the side effects of smoking, such as having yellow teeth, having wrinkles on face in a very early age, or having bad breathe? \| 1= Yes 2= No \| R2 \| \| 3 \| **Have you heard about the following non-communicable diseases?** \| \| R3 \| \| Diabetes \| 1= Yes 2= No \| R3a \| \| Hypertension \| 1= Yes 2= No \| R3b \| \| Heart diseases \| 1= Yes 2= No \| R3c \| \| Stroke \| 1= Yes 2= No \| R3d \| \| Lung cancer \| 1= Yes 2= No \| R3e \| \| Stomach cancer \| 1= Yes 2= No \| R3f \| \| Liver diseases \| 1= Yes 2= No \| R3g \| \| Tuberculosis \| 1= Yes 2= No \| R3h \| \| 4 \| **Do you know smoking cigarette causes the following diseases?** \| \| R4 \| \| Diabetes \| 1= Yes 2= No \| R4a \| \| Hypertension \| 1= Yes 2= No \| R4b \| \| Heart diseases \| 1= Yes 2= No \| R4c \| \| Stroke \| 1= Yes 2= No \| R4d \| \| Lung cancer \| 1= Yes 2= No \| R4e \| \| Stomach cancer \| 1= Yes 2= No \| R4f \| \| Liver diseases \| 1= Yes 2= No \| R4g \| \| Tuberculosis \| 1= Yes 2= No \| R4h \| \| 5 \| **Do you know high salt intake causes the following diseases?** \| \| R5 \| \| Diabetes \| 1= Yes 2= No \| R5a \| \| Hypertension \| 1= Yes 2= No \| R5b \| \| Heart diseases \| 1= Yes 2= No \| R5c \| \| Stroke \| 1= Yes 2= No \| R5d \| \| 6 \| **Do you know unhealthy diet such as eating soft drinks, fast food, or high red meat intake causes the following diseases?** \| \| R5 \| \| Diabetes \| 1= Yes 2= No \| R5a \| \| Hypertension \| 1= Yes 2= No \| R5b \| \| Heart diseases \| 1= Yes 2= No \| R5c \| \| Stroke \| 1= Yes 2= No \| R5d \| \| 7 \| **Do you know less physical activity cause the following diseases?** \| \| R6 \| \| Diabetes \| 1= Yes 2= No \| R6a \| \| Hypertension \| 1= Yes 2= No \| R6b \| \| Heart diseases \| 1= Yes 2= No \| R6e \| \| Stroke \| 1= Yes 2= No \| R6f \| \| 8 \| **Do you know overweight/obese cause the following diseases?** \| \| R7 \| \| Diabetes \| 1= Yes 2= No \| R7a \| \| Hypertension \| 1= Yes 2= No \| R7b \| \| Heart diseases \| 1= Yes 2= No \| R7c \| \| Stroke \| 1= Yes 2= No \| R7d \| |
| --- | --- | --- | --- | --- | --- | --- | --- | --- | --- | --- | --- | --- | --- | --- | --- | --- | --- | --- | --- | --- | --- | --- | --- | --- | --- | --- | --- | --- | --- | --- | --- | --- | --- | --- | --- | --- | --- | --- | --- | --- | --- | --- | --- | --- | --- | --- | --- | --- | --- | --- | --- | --- | --- | --- | --- | --- | --- | --- | --- | --- | --- | --- | --- | --- | --- | --- | --- | --- | --- | --- | --- | --- | --- | --- | --- | --- | --- | --- | --- | --- | --- | --- | --- | --- | --- | --- | --- | --- | --- | --- | --- | --- | --- | --- | --- | --- | --- | --- | --- | --- | --- | --- | --- | --- | --- | --- | --- | --- | --- | --- | --- | --- | --- | --- | --- | --- | --- | --- | --- | --- | --- | --- | --- | --- | --- | --- | --- | --- | --- | --- | --- | --- | --- | --- | --- | --- |

| \| **Step 7 Anthropometric measurements** \| \| \| \| \| --- \| --- \| --- \| --- \| \| 1 \| Height \| Cm: \| A1 \| \| 2 \| Weight \| Kg: \| A2 \| |
| --- | --- | --- | --- | --- | --- | --- | --- | --- | --- | --- | --- | --- |

**Table S1:** Multivariable-adjusted odds ratio (OR) and 95% confidence interval (CI) for the association of total sleep duration with underweight and overweight/obesity

|  | Under weight | | Overweight/obesity | |
| --- | --- | --- | --- | --- |
|  | OR | (95% CI) | OR | (95% CI) |
| **Total sleep duration (hours/day)** | |  |  |  |
| ≥8 | 1.00 (reference) |  | 1.00 (reference) |  |
| 7 to <8 | 0.76 | (0.50 - 1.15) | 1.10 | (0.76 - 1.60) |
| <7 | 0.33 | (0.20 - 0.54) | 1.73 | (1.21 - 2.47) |
| **Age (Years)** | 1.60 | (1.31 - 1.94) | 0.74 | (0.63 - 0.88) |
| **Sex** |  |  |  |  |
| Boys | 1.00 (reference) |  | 1.00 (reference) |  |
| Girls | 0.88 | (0.61- 1.28) | 1.09 | (0.80 - 1.47) |
| **Number of household members** | 1.18 | (1.04 - 1.34) | 0.78 | (0.67 - 0.91) |
| **Soft drink consumption** | |  |  |  |
| Less than once/week | 1.00 (reference |  | 1.00 (reference |  |
| once/week | 0.62 | (0.35 -1.11) | 1.00 | (0.65 - 1.56) |
| ≥2 times/week | 0.77 | (0.47 - 1.28) | 0.83 | (0.56 - 1.25) |
| **Fast food consumption** | |  |  |  |
| Less than once/week | 1.00 (reference |  | 1.00 (reference |  |
| Once/week | 1.02 | (0.65 -1.60) | 0.89 | (0.62 - 1.28) |
| ≥2 times/week | 1.06 | (0.67-1.68) | 0.79 | (0.54 - 1.15) |
| **General physical activities** | |  |  |  |
| <30 minutes/day | 1.00 (reference |  | 1.00 (reference |  |
| 30-59 minutes/day | 0.62 | (0.35 - 1.11) | 1.20 | (0.79 - 1.82) |
| ≥60 minutes/day | 0.99 | (0.67- 1.45) | 1.18 | (0.86 - 1.63) |
| **Sitting activities** |  |  |  |  |
| <5 hours a day | 1.00 (reference |  | 1.00 (reference |  |
| ≥ 5 hours a day | 0.77 | (0.43 - 1.40) | 1.39 | (0.87 - 2.22) |
| **Exposure to passive smoking** | |  |  |  |
| 0 days | 1.00 (reference |  | 1.00 (reference |  |
| 1-2 days | 0.98 | (0.64 - 1.51) | 1.22 | (0.86 - 1.73) |
| 3-4 days | 1.15 | (0.71 - 1.88) | 0.78 | (0.50 -1.23) |
| ≥5 days | 0.57 | (0.28 -1.17) | 1.07 | (0.67 - 1.73) |

**Table S2:** Multivariable-adjusted odds ratio (OR) and 95% confidence interval (CI) for the association of weekday sleep duration with underweight and overweight/obesity

|  | Under weight | | Overweight/obesity | |
| --- | --- | --- | --- | --- |
|  | OR | (95% CI) | OR | (95% CI) |
| **Weekday sleep duration (hours/day)** | |  |  |  |
| ≥8 | 1.00 (reference) |  | 1.00 (reference) |  |
| 7 to <8 | 0.77 | (0.49 - 1.21) | 1.17 | (0.77 - 1.78) |
| <7 | 0.55 | (0.35 - 0.84) | 1.43 | (0.98 - 2.11) |
| **Age (Years)** | 1.59 | (1.31 - 1.94) | 0.74 | (0.63 - 0.88) |
| **Sex** |  |  |  |  |
| Boys | 1.00 (reference) |  | 1.00 (reference) |  |
| Girls | 0.90 | (0.62- 1.30) | 1.07 | (0.79 - 1.45) |
| **Number of household members** | 1.19 | (1.05 - 1.36) | 0.78 | (0.68 - 0.91) |
| **Soft drink consumption** | |  |  |  |
| Less than once/week | 1.00 (reference |  | 1.00 (reference |  |
| Once/week | 0.66 | (0.37 -1.17) | 0.98 | (0.63 - 1.51) |
| ≥2 times/week | 0.82 | (0.50 - 1.35) | 0.81 | (0.54 - 1.21) |
| **Fast food consumption** | |  |  |  |
| Less than once/week | 1.00 (reference |  | 1.00 (reference |  |
| Once/week | 1.10 | (0.70-1.72) | 0.85 | (0.60 - 1.22) |
| ≥2 times/week | 1.09 | (0.69-1.72) | 0.79 | (0.54 - 1.14) |
| **General physical activities** | |  |  |  |
| <30 minutes/day | 1.00 (reference |  | 1.00 (reference |  |
| 30-59 minutes/day | 0.65 | (0.36 - 1.15) | 1.17 | (0.78 - 1.77) |
| ≥60 minutes/day | 1.07 | (0.73- 1.56) | 1.14 | (0.83 - 1.58) |
| **Sitting activities** |  |  |  |  |
| <5 hours a day | 1.00 (reference |  | 1.00 (reference |  |
| ≥ 5 hours a day | 0.79 | (0.44 - 1.42) | 1.38 | (0.87 - 2.21) |
| **Exposure to passive smoking** | |  |  |  |
| 0 days | 1.00 (reference |  | 1.00 (reference |  |
| 1-2 days | 1.00 | (0.65 - 1.53) | 1.21 | (0.85 - 1.71) |
| 3-4 days | 1.17 | (0.72 - 1.89) | 0.79 | (0.50 -1.23) |
| ≥5 days | 0.56 | (0.28 -1.15) | 1.08 | (0.67 - 1.74) |

**Table S3:** Multivariable-adjusted odds ratio (OR) and 95% confidence interval (CI) for the association of weekend sleep duration with underweight and overweight/obesity

|  | Under weight | | Overweight/obesity | |
| --- | --- | --- | --- | --- |
|  | OR | (95% CI) | OR | (95% CI) |
| **Week end sleep duration (hours/day)** | |  |  |  |
| ≥8 | 1.00 (reference) |  | 1.00 (reference) |  |
| 7 to <8 | 0.79 | (0.50 - 1.25) | 1.36 | (0.95 - 1.96) |
| <7 | 0.53 | (0.31 - 0.89) | 1.46 | (1.00 - 2.12) |
| **Age (Years)** | 1.60 | (1.32 - 1.95) | 0.74 | (0.63 - 0.88) |
| **Sex** |  |  |  |  |
| Boys | 1.00 (reference) |  | 1.00 (reference) |  |
| Girls | 0.86 | (0.60- 1.24) | 1.08 | (0.80 - 1.46) |
| **Number of household members** | 1.20 | (1.05 - 1.36) | 0.78 | (0.67 - 0.91) |
| **Soft drink consumption** | |  |  |  |
| Less than once/week | 1.00 (reference |  | 1.00 (reference |  |
| Once/week | 0.66 | (0.38 -1.18) | 0.99 | (0.64 - 1.52) |
| ≥2 times/week | 0.83 | (0.50 - 1.36) | 0.82 | (0.55 - 1.23) |
| **Fast food consumption** | |  |  |  |
| Less than once/week | 1.00 (reference |  | 1.00 (reference |  |
| Once/week | 1.06 | (0.68-1.67) | 0.86 | (0.60 - 1.23) |
| ≥2 times/week | 1.05 | (0.67-1.67) | 0.81 | (0.56 - 1.17) |
| **General physical activities** | |  |  |  |
| <30 minutes/day | 1.00 (reference |  | 1.00 (reference |  |
| 30-59 minutes/day | 0.63 | (0.35 - 1.12) | 1.20 | (0.79 - 1.82) |
| ≥60 minutes/day | 1.04 | (0.71- 1.52) | 1.17 | (0.85 - 2.17) |
| **Sitting activities** |  |  |  |  |
| <5 hours a day | 1.00 (reference |  | 1.00 (reference |  |
| ≥ 5 hours a day | 0.81 | (0.45 - 1.46) | 1.36 | (0.85 - 2.17) |
| **Exposure to passive smoking** | |  |  |  |
| 0 days | 1.00 (reference |  | 1.00 (reference |  |
| 1-2 days | 1.01 | (0.66 - 1.54) | 1.21 | (0.86 - 1.71) |
| 3-4 days | 1.15 | (0.71 - 1.86) | 0.82 | (0.52 -1.29) |
| ≥5 days | 0.55 | (0.27 -1.13) | 1.12 | (0.69 - 1.79) |

**Table S4:** Multivariable-adjusted odds ratio and 95% confidence interval for the association of sleep duration with underweight and overweight/obesity

|  | **Underweight** | | **Overweight/obesity** | |
| --- | --- | --- | --- | --- |
|  | Model 1^a^ | Model 2^b^ | Model 1^a^ | Model 2^b^ |
| **Weekday night sleep duration (hours/day)** | |  |  |  |
| ≥8 | 1.00 (reference) | 1.00 (reference) | 1.00 (reference) | 1.00 (reference) |
| 7 to <8 | 0.77 (0.49-1.21) | 0.77 (0.49-1.21) | 1.19 (0.79-1.80) | 1.17 (0.77-1.78) |
| <7 | 0.54 (0.35-0.82) | 0.55 (0.35-0.84) | 1.43 (0.98-2.09) | 1.43 (0.98-2.11) |
| *P_trend_* | 0.004 | 0.006 | 0.056 | 0.058 |
| **Weekend night sleep duration (hours/day)** | |  |  |  |
| ≥8 | 1.00 (reference) | 1.00 (reference) | 1.00 (reference) | 1.00 (reference) |
| 7 to <8 | 0.79 (0.50-1.23) | 0.79 (0.50-1.25) | 1.42 (0.99-2.02) | 1.36 (0.95-1.96) |
| <7 | 0.53 (0.32-0.89) | 0.53 (0.31-0.89) | 1.48 (1.03-2.12) | 1.46 (1.00-2.12) |
| *P_trend_* | 0.014 | 0.015 | 0.018 | 0.029 |

^a^Model 1 adjusted for age (year, continuous) and sex (boys or girls)

^b^Model 2 variables adjusted in model 1 + number of household members (continuous), soft drink consumption (less than once/week, once/week, ≥2 times/week), fast food consumption (less than once/week, once/week, ≥2 times/week), general physical activity (<30 minutes/day, 30-59 minutes/day, ≥60 minutes/day), sitting activities (<5 hours a day, ≥ 5 hours a day), and exposure to passive smoking (0 days, 1-2 days, 3-4 days, ≥5 days)

**Table S5:** Association between the study covariates and BMI

|  | **BMI** | | | **p-value^a^** |
| --- | --- | --- | --- | --- |
|  | Underweight  n (%) | Normal  n (%) | Overweight/obesity  n (%) |  |
| **Age (**Mean±SD) | 14.25±0.87 | 13.85±0.93 | 13.64 ±0.89 | 0.0001 |
| **Sex** |  |  |  | 0.198 |
| Boy | 85 (54.84) | 300 (47.92) | 120 (45.98) |  |
| Girl | 70 (45.16) | 326 (52.08) | 141 (54.02) |  |
| **Number of household members (**Mean±SD) | 4.97±1.30 | 4.71±1.25 | 4.43±1.01 | 0.0001 |
| **Soft drink consumption** |  |  |  | 0.093 |
| Less than once/week | 28 (18.06) | 98 (15.65) | 53 (20.31) |  |
| Once/week | 32 (20.65) | 159 (25.40) | 76 (29.12) |  |
| ≥2 times/week | 95 (61.29) | 369 (58.95) | 132 (50.57) |  |
| **Fast food consumption** |  |  |  | 0.616 |
| Less than once/week | 48 (30.97) | 192 (30.67) | 93 (35.63) |  |
| Once/week | 51 (32.90) | 209 (33.39) | 86 (32.95) |  |
| ≥2 times/week | 56 (36.13) | 225 (35.94) | 82 (31.42) |  |
| **General physical activity** |  |  |  | 0.287 |
| <30 minutes/day | 73 (47.10) | 291 (46.49) | 112 (42.91) |  |
| 30-59 minutes/day | 17 (10.97) | 107 (17.09) | 46 (17.62) |  |
| ≥60 minutes/day | 65 (41.94) | 228 (36.42) | 103 (39.46) |  |
| **Sitting activities** |  |  |  | 0.816 |
| <5 hours a day | 139 (89.68) | 563 (89.94) | 231 (88.51) |  |
| ≥ 5 hours a day | 16 (10.32) | 63 (10.06) | 30 (11.49) |  |
| **Exposure to passive smoking** |  |  |  | 0.182 |
| 0 days | 73 (47.10) | 294 (46.96) | 122 (46.74) |  |
| 1-2 days | 42 (27.10) | 152 (24.28) | 75 (28.74) |  |
| 3-4 days | 30 (19.35) | 105 (16.77) | 32 (12.26) |  |
| ≥5 days | 10 (6.45) | 75 (11.98) | 32 (12.26) |  |

^a^Based on Kruskal Wallis test for continuous variables and chi-square test for categorical variables.
